# HIV incidence among women engaging in sex work in sub-Saharan Africa: a systematic review and meta-analysis

**DOI:** 10.1016/S2214-109X(24)00227-4

**Published:** 2024-07-17

**Authors:** Harriet S Jones, Rebecca L Anderson, Henry Cust, R Scott McClelland, Barbra A Richardson, Harsha Thirumurthy, Kalonde Malama, Bernadette Hensen, Lucy Platt, Brian Rice, Frances M Cowan, Jeffrey W Imai-Eaton, James R Hargreaves, Oliver Stevens

**Affiliations:** a Faculty of Public Health and Policy, London School of Hygiene and Tropical Medicine, London, UK; b MRC Centre for Global Infectious Disease Analysis, School of Public Health, Imperial College London, London, UK; c Institute of Global Health, University College London, London, UK; d Department of Medicine, University of Washington, Seattle, WA, USA; e Department of Epidemiology, University of Washington, Seattle, WA, USA; f Department of Biostatistics, University of Washington, Seattle, WA, USA; g Department of Global Health, University of Washington, Seattle, WA, USA; h Department of Medical Ethics and Health Policy, University of Pennsylvania, Philadelphia, PA, USA; i Ingram School of Nursing, McGill University, Montréal, Quebec, QC, Canada; j Sexual and Reproductive Health Group, Department of Public Health, Institute of Tropical Medicine, Antwerp, Belgium; k Sheffield Centre for Health and Related Research, School of Medicine and Population Health, University of Sheffield, UK; l Department of International Public Health, Liverpool School of Tropical Medicine, Liverpool, UK; m Centre for Sexual Health and HIV/AIDS Research Zimbabwe, Harare, Zimbabwe; n Center for Communicable Disease Dynamics, Department of Epidemiology, Harvard T H Chan School of Public Health, Boston, MA, USA

## Abstract

**Background:**

Women who engage in sex work in sub-Saharan Africa have a high risk of acquiring HIV infection. HIV incidence has declined among all women in sub-Saharan Africa, but trends among women who engage in sex work are poorly characterised. We synthesised data on HIV incidence among women who engage in sex work in sub-Saharan Africa and compared these with the total female population to understand relative incidence and trends over time.

**Methods:**

We searched MEDLINE, Embase, Global Health, and Google Scholar from Jan 1, 1990, to Feb 28, 2024, and grey literature for studies that reported empirical estimates of HIV incidence among women who engage in sex work in any sub-Saharan Africa country. We calculated incidence rate ratios (IRRs) compared with total female population incidence estimates matched for age, district, and year, did a meta-analysis of IRRs, and used a continuous mixed-effects model to estimate changes in IRR over time.

**Findings:**

From 32 studies done between 1985 and 2020, 2194 new HIV infections were observed among women who engage in sex work over 51 490 person-years. Median HIV incidence was 4·3 per 100 person years (IQR 2·8–7·0 per 100 person-years). Incidence among women who engage in sex work was eight times higher than matched total population women (IRR 7·8 [95% CI 5·1–11·8]), with larger relative difference in western and central Africa (19·9 [9·6–41·0]) than in eastern and southern Africa (4·9 [3·4–7·1]). There was no evidence that IRRs changed over time (IRR per 5 years: 0·9 [0·7–1·2]).

**Interpretation:**

Across sub-Saharan Africa, HIV incidence among women who engage in sex work remains disproportionately high compared with the total female population. However, constant relative incidence over time indicates HIV incidence among women who engage in sex work has declined at a similar rate. Location-specific data for women who engage in sex work incidence are sparse, but improved surveillance and standardisation of incidence measurement approaches could fill these gaps. Sustained and enhanced HIV prevention for women who engage in sex work is crucial to address continuing inequalities and ensure declines in new HIV infections.

**Funding:**

Bill & Melinda Gates Foundation, UK Research and Innovation, National Institutes of Health.

**Translation:**

For the French translation of the abstract see Supplementary Materials section.

## Introduction

Among women in sub-Saharan Africa, women who engage in sex work are disproportionately affected by HIV.^1^, ^2^ Women who engage in sex work comprise an estimated 1·2% of women aged 15–49 years in sub-Saharan Africa, but an estimated 3·5% of women living with HIV in the region.^2^ Despite evidence of substantially higher HIV prevalence,^2^, ^3^, ^4^ HIV incidence among women who engage in sex work is poorly characterised. HIV prevention programmes for women who engage in sex work were a core component of the early HIV response in sub-Saharan Africa; however, reduced funding and a shift to general population programming reduced programmes focused on women who engage in sex work from the early 2000s.^5^ More recently, renewed attention on focused approaches for key populations has re-expanded programming for women who engage in sex work.^6^, ^7^, ^8^ Assessing the success of these efforts at preventing new HIV infections is challenging. Although new HIV infections have steadily declined among all women in sub-Saharan Africa,^1^ whether HIV incidence among women who engage in sex work has declined at a similar rate is unknown.

Despite increasing surveillance and programmes for women who engage in sex work, studies of HIV incidence in sub-Saharan Africa remain infrequent and challenging. Identifying and following up women who engage in sex work is often difficult due to the heterogeneous, informal, and hidden nature of sex work, commonly driven by stigma and criminalisation.^9^ For these reasons, women who engage in sex work are largely unidentified in population-based household surveys, and constructing a representative national sampling frame for women who engage in sex work is impractical. Sex work encompasses a broad spectrum of sexual transactions occurring in multiple settings, from on streets, to in homes, brothels, or hotels.^10^ Women who exchange sex for money and goods might not self-identify as sex workers; for surveillance, this presents challenges, as those not identifying as sex workers are unlikely to present at sex worker dedicated programmes. Additionally, mobility among women who engage in sex work is high,^11^ and repeated initiation and cessation of sex work common, precipitating commonly high loss to follow-up for programmes and cohort studies.^12^, ^13^ To mitigate these challenges, methods have been developed leveraging cross-sectional data to estimate incidence,^14^, ^15^ and recruitment approaches such as respondent-driven sampling and time-location sampling are increasingly used to capture more representative samples of women who engage in sex work.

**Research in context**

**Evidence before this study**

Women who engage in sex work are disproportionately affected by HIV across sub-Saharan Africa, comprising an estimated 1·2% of woman aged 15–49 in the region, but an estimated 3·5% of women living with HIV. Empirical estimates of HIV incidence among women who engage in sex work are limited, with individual studies often narrow in geographical scope and rarely repeated in the same populations. Despite overall declines in HIV incidence among all women in sub-Saharan Africa, it is unclear whether incidence among women who engage in sex work is declining at a similar rate. Previous efforts to synthesise data on HIV incidence among women who engage in sex work have assessed global differences in incidence by age, with limited focus on sub-Saharan Africa or on changes in incidence over time.

**Added value of this study**

We systematically searched and meta-analysed studies published between Jan 1, 1990 and Feb 28, 2024 reporting empirical measurements of HIV incidence among women who engage in sex work in sub-Saharan Africa. Our review identified a greater number of empirical estimates and substantially increased the geographical reach and time period covered by any previous review of HIV incidence among women who engage in sex work in sub-Saharan Africa. Our analysis quantified the discrepancy between incidence in women who engage in sex work and total population women, estimating rates among women who engage in sex work to be 7·8 times higher (incidence rate ratio 7·8, 95% CI 5·1–11·8), with a greater disparity in western and central Africa compared with eastern and southern Africa. Our work addresses methodological challenges synthesising heterogeneous studies by accounting for the geographical and age variation in recruited populations. The review contributes to improving characterisation of HIV incidence among women who engage in sex work in the region by assessing trends over time and situating these findings within the context of the HIV epidemic among age-matched women in the wider population.

**Implications of all the available evidence**

Our study underscores the need to continue expansion of effective prevention and treatment programming for women who engage in sex work. Our study also highlights the geographical and methodological gaps that remain in surveillance activities and the need to ensure a more standardised approach to obtaining empirical estimates of incidence among women who engage in sex work. Improved incidence measurement will strengthen and guide data-driven HIV programming. Quantifying and broadening our understanding of the inequalities that persist in the HIV epidemic provides a practical assessment for programmatic need and progress towards targets to be reached through continued and sustained HIV prevention.

As countries seek to reach the global goal of ending AIDS as a public health threat by 2030 through ending inequalities, quantifying HIV incidence trends among women who engage in sex work is required to guide national HIV programme planning and delivery. In this study, we aimed to synthesise and appraise empirical estimates of HIV incidence in women who engage in sex work in sub-Saharan Africa, estimate relative HIV incidence with the total female population for western and central Africa and eastern and southern Africa, and estimate the change in HIV incidence over time among women who engage in sex work, relative to incidence trends among all women.

## Methods

### Study design

We searched published and grey literature to identify empirical estimates of HIV incidence among women who engage in sex work in sub-Saharan Africa. We conducted our searches with no language restrictions for peer-reviewed literature published between Jan 1, 1990, and Feb 28, 2024, and replicated searches in French to identify any additional publications. Our initial screening was from a preliminary search of papers identified through an earlier review of HIV testing among women who engage in sex work.^16^ MEDLINE, Embase, POPLINE, Web of Science, and Global Health were searched on June 19, 2019, by authors HSJ and HC using medical subject headings and text words adapted for each database covering three domains: “female sex workers”, “HIV”, and “sub-Saharan Africa”. Full texts were subsequently searched for those reporting HIV incidence by HSJ. An updated search and screening was conducted by RLA on Feb 28, 2024 in MEDLINE, Embase, Global Health, and Google Scholar using text words addressing four domains: “sex workers”, “sub-Saharan Africa”, “HIV”, and “incidence” (appendix 2 p 4). RLA checked that the second search included all papers identified in the initial search. RLA additionally searched key population biobehavioural surveillance reports (grey literature), which was collated during an earlier key population data collation exercise.^2^ All papers were selected for final inclusion based on consensus between authors HSJ, RLA, and OS.

This study received ethical approval from the Imperial College Research and Ethics Committee (6412027). For use of the Centre for Sexual Health and HIV/AIDS Research Zimbabwe (CeSHHAR) Key Populations programme data, ethical approval was obtained from the London School of Hygiene and Tropical Medicine (16543) and the Medical Research Council of Zimbabwe (MRCZ/A/2624).

### Study inclusion

**Figure 1:**
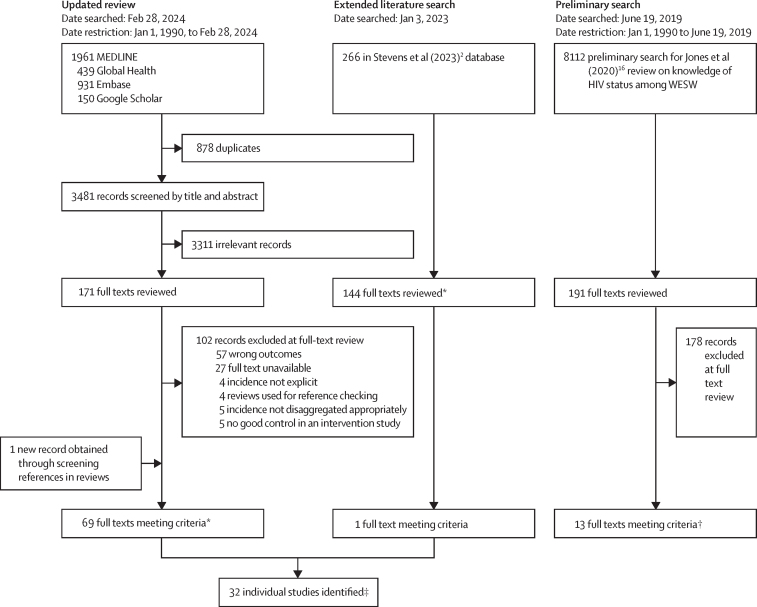
Trial profile WESW=women who engage in sex work. *Full texts meeting criteria include multiple papers reporting on the same study. †All papers identified in the preliminary search were identified in the updated review. Individual studies identified are the primary texts from which data were extracted from.

Included studies were required to report an empirically measured estimate of serologically confirmed HIV incidence among women who engage in sex work in any sub-Saharan Africa country or report the number of HIV events and total person-years at-risk to calculate incidence. Women who engage in sex work could be cisgender or transgender women either self-reporting being a sex worker, engaged in a sex worker programme, or reporting selling or exchanging sex for money or goods. For multicountry studies, nationally disaggregated data were required. For closed cohort studies reporting annual estimates, only the overall estimate of incidence from the study was extracted to avoid artificial declines in observed incidence with follow-up of the same individuals. Studies were excluded if the study cohort was exposed to an intervention specifically intended to affect HIV incidence—eg, HIV pre-exposure prophylaxis. Data from the control group in randomised control trials and from early or pre-intervention periods in interventional cohort studies were included, as these were likely to be more representative of the wider population of women who engage in sex work from which data were collected. Conference abstracts or published short communications were excluded. When multiple papers reported incidence estimates for the same study population, the paper reporting the greatest number of person-years of follow-up was selected as the primary study (figure 1).

### Procedures

From each study, two authors extracted the study location (country, subnational area), time period, study population definition, recruitment strategy, mean or median age and eligible age range, incidence measurement method (eg, repeat HIV tests, recent infection testing), study type (eg, cohort, randomised controlled trial), sample size, person-years of follow-up, number of seroconversions, incidence rate per 100 person-years, and CIs, disaggregated by age-group when available. We contacted study authors when estimates were not clearly available in existing publications (n=1),^17^ not disaggregated to women who engage in sex work (n=1),^18^ or from studies reporting data for extended time periods that could provide annual estimates to assess temporal trends (n=2).^19^, ^20^

### Quality assessment

We appraised studies using the Global HIV Quality Assessment Tool for Data Generated through Non-Probability Sampling (GHQAT).^21^ The tool comprises three domains: study design, study implementation, and a measurement specific domain for HIV incidence. Domain-specific assessment criteria are in the appendix 2 (p 10). A score of 1 was assigned if studies met the assessment criteria and 0 if they did not. Each study was classified as good, fair, or poor for each domain, and then overall. Scoring was agreed between HSJ, RLA, and OS based on the narrative assessment findings. Studies scoring 70% or more were classified as good, 30–70% as fair, and less than 30% as poor. We excluded assessment criteria on follow-up time and participant retention when these were not relevant (ie, for cross-sectional estimates of HIV incidence using recent HIV infection testing or HIV prevalence).

### Data synthesis and meta-analysis

HIV incidence observations among women who engage in sex work were matched to HIV incidence in the total female population by district (subnational location where each study was conducted), age, and year. Estimates for missing age information were matched to ages 15–39 years, reflecting the age distribution observed in women who engage in sex work in studies that reported age data. District-level estimates for the total female population in 2022 were extracted from UNAIDS subnational HIV estimates created using the Naomi small-area estimation model.^22^ District-level incidence estimates for 1985–2021 were created by extrapolating 2022 estimates backwards in time parallel to age-matched UNAIDS national-level female incidence trajectories, assuming the proportional change in incidence at district level mirrored that at national level.^23^, ^24^ For studies without annual incidence estimates, the midpoint study year was used for matching. When a subnational location was not specified in the text (n=3), national total population incidence was used as the comparator. One study reported incidence for a control group of women not engaged in sex work,^25^ which was used as the total population comparator instead of matching to UNAIDS’ district estimates.

We assessed correlation between women who engage in sex work and matched total population female incidence and calculated incidence rate ratios (IRRs). We meta-analysed IRRs, with study-district nested random effects to account for variation in study type, and regional fixed effects to stratify the pooled IRRs by region (eastern and southern Africa and western and central Africa). The *I*^2^ statistic was used to assess heterogeneity. Due to high heterogeneity, we also calculated median IRRs with IQRs by region. We estimated time trends in log IRRs with a Bayesian mixed-effects log-linear model. The regression model included a linear trend for calendar year, and study-level random effects. Log total female population incidence and person-years of follow-up were specified as offsets such that model coefficients reflected log IRRs relative to matched population female incidence (appendix 2 p 6). We also conducted case studies for the two countries with the most available data. We used data from an open cohort in Mombasa, Kenya^19^ and from a national key populations programme in Zimbabwe run by the Centre for Sexual Health and HIV/AIDS Research Zimbabwe (CeSHHAR),^20^ to descriptively assess temporal trends between women who engage in sex work and total female population incidence.

We conducted sensitivity analyses. To address uncertainty about district-level total population incidence estimates we repeated the meta-analysis using national age–sex matched population incidence as the denominator for IRRs. To assess the effect of study quality, we repeated the meta-analysis restricted to higher quality studies (those scoring 60% or above on the GHQAT). Finally, as the majority of empirical incidence estimates for women who engage in sex work were from populations in Kenya and Zimbabwe, we refit the mixed-effects model to data from both countries. Analyses were implemented in R v4.2.1 using the *metafor* v3.8.1^26^ and *R-INLA* v22.5.7^27^ packages.

### Role of the funding source

The funders of the study had no role in study design, data collection, data analysis, data interpretation, or writing of the report.

## Results

We extracted 83 estimates of HIV incidence among women who engage in sex work in sub-Saharan Africa from 32 studies reported in 69 peer-reviewed papers and one surveillance report (Table 1, Table 2). 65 (78%) of 83 estimates were from eastern and southern Africa, predominantly from Kenya and Zimbabwe (49 [59%] of 83). In western and central Africa, 18 incidence estimates were reported from eight countries. Median study year was 2008 (IQR 2000–15). Between 1985 and 2020, 2194 new HIV infections were observed from 51 490 person years with median HIV incidence of 4·3 per 100 person years (IQR 2·8–7·0 per 100 person years; eastern and southern Africa: 4·3 per 100 person years [2·9–6·3]; western and central Africa: 3·8 per 100 person years [2·2–7·5]).

**Table 1:** Annual incidence estimates for women who engage in sex work and women from the general population matched for area, year, and age

	**Study period**	Study location*	**Midpoint year**	**Age group (years)**†	**Women who engage in sex work new HIV infections**	**Person years**	**Women who engage in sex work incidence per 100 person years**	**Total female population incidence per 100 person years**‡	**IRR**§
**Ali et al (2022)**^15^§									
Zimbabwe	2011–17	Zimbabwe	2014	15–24	7	105	6·3 (5·3–7·6)	1·07	5·90
Zimbabwe	2011–17	Zimbabwe	2014	25–39	6	175	3·3 (1·3–4·2)	0·88	3·74
**Botswana Ministry of Health (2013)**^28^¶									
Botswana	2012	Gaborone–Francistown–Kasane	2012	15–39	46	450	12·5 (7·3–17·1)	1·80	6·93
**Braunstein et al (2011)**^29^									
Rwanda	2006–08	Kigali	2007	15–39	3	91	3·3 (0·0–7·0)	0·47	7·05
**Chabata et al (2021)**^30^									
Zimbabwe	2017–19	Chinhoyi	2018	15–24	16	226	7·1 (4·3–11·5)	0·49	14·39
Zimbabwe	2017–19	Karoi	2018	15–24	11	193	5·7 (3·2–10·3)	0·46	12·44
Zimbabwe	2017–19	Kwekwe	2018	15–24	12	278	4·3 (2·5–7·6)	0·69	6·29
Zimbabwe	2017–19	Zvishavane	2018	15–24	9	210	4·3 (2·2–8·2)	0·65	6·54
**Chersich et al (2014)**^31^									
Kenya	2006–07	Mombasa (Kisauni and Chaani)	2007	15–39	10	381	2·6	0·62	4·20
**Diabaté et al (2018)**^32^									
Benin	2008–12	Cotonou	2010	15–39	6	425	1·4 (0·3–2·5)	0·33	4·26
**Faini et al (2022)**^33^									
Tanzania	2018–19	Dar es Salaam	2019	15–24	12	278	4·3	0·05	80·58
Tanzania	2018–19	Dar es Salaam	2019	25–34	5	234	2·1	0·07	32·60
Tanzania	2018–19	Dar es Salaam	2019	35–44	4	97	4·1	0·05	79·31
**Forbi et al (2011)**^34^									
Nigeria	2006	Nasarawa State	2006	15–34	71	590	12·0 (8·5–15·4)	0·47	25·53
**Fowke et al (1996)**^35^									
Kenya	1985–94	Nairobi	1990	15–39	239	569	42·0	3·21	13·07
**Ghys et al (2001)**^36^									
Côte d'Ivoire	1994–98	Abidjan	1996	15–39	11	68	16·3	2·07	7·88
**Gilbert et al (2003)**^37^									
Senegal	1985–99	Dakar	1987	15–24	34	1628	2·1	0·01	235·63
Senegal	1985–99	Dakar	1987	25–34	91	5474	1·7	0·02	82·60
Senegal	1985–99	Dakar	1987	35–44	58	1939	3·0	0·01	216·60
Senegal	1985–99	Dakar	1987	45–49	12	260	4·6	0·01	639·76
**Jones et al (2023)**^20^‖									
Zimbabwe	2010–19	Zimbabwe	2010	15–39	3	56	5·4 (3·2–10·9)	1·28	4·20
Zimbabwe	2010–19	Zimbabwe	2011	15–39	6	139	4·3 (2·2–14·6)	1·20	3·60
Zimbabwe	2010–19	Zimbabwe	2012	15–39	15	264	5·7 (1·9–22·8)	1·11	5·10
Zimbabwe	2010–19	Zimbabwe	2013	15–39	12	410	2·9 (1·5–8·1)	1·04	2·81
Zimbabwe	2010–19	Zimbabwe	2014	15–39	33	731	4·5 (2·5–8·2)	0·97	4·66
Zimbabwe	2010–19	Zimbabwe	2015	15–39	62	1402	4·4 (3·6–5·4)	0·89	4·99
Zimbabwe	2010–19	Zimbabwe	2016	15–39	58	1857	3·1 (2·5–4·1)	0·79	3·98
Zimbabwe	2010–19	Zimbabwe	2017	15–39	94	2422	3·9 (3·3–4·7)	0·68	5·69
Zimbabwe	2010–19	Zimbabwe	2018	15–39	114	2946	3·9 (3·3–4·7)	0·55	7·02
Zimbabwe	2010–19	Zimbabwe	2019	15–39	44	1432	3·1 (2·1–5·0)	0·47	6·56
**Kasamba et al (2019)**^38^									
Uganda	Cohort 1: 2008–17; cohort 2: 2013–17	Kampala	2010	15–39	59	2007	2·9	1·15	2·53
Uganda	Cohort 1: 2008–17; cohort 2: 2013–17	Kampala	2014	15–39	46	1394	3·3	0·93	3·53
Uganda	Cohort 1: 2008–17; cohort 2: 2013–17	Kampala	2016	15–39	65	2138	3·0	0·84	3·59
**Kassanjee et al (2022)**^14^¶									
South Africa	2019	South Africa	2019	15–49	190	4130	4·6 (1·5–8·5)	1·21	3·81
**Kaul et al (2004)**^39^									
Kenya	1998–2002	Nairobi	2000	15–39	16	495	3·2	1·29	2·47
**Kerrigan et al (2019)**^40^									
Tanzania	2015–17	Mafinga	2002	15–39	10	144	6·9	2·54	2·73
**Kilburn et al (2018)**^25^									
South Africa	2012–15	Mpumalanga	2014	15–19	41	1273	3·2	1·85	1·74
**Laga et al (1994)**^41^									
Democratic Republic of Congo	1988–91	Kinshasa	1988	15–39	70	880	11·7	0·34	34·68
**Lyons et al (2020)**^42^									
Senegal	2015–17	Dakar–Mbour–Theis	2016	15–39	4	303	1·3 (0·5–3·5)	0·01	161·67
**Malama et al (2022)**^17^									
Zambia	2012–17	Ndola–Lusaka	2015	15–44	24	884	2·7	1·84	1·49
**McClelland et al (2015)**^19^‖									
Kenya	1993–2017	Mombasa	1993	15–39	20	335	6·0	3·24	1·84
Kenya	1993–2017	Mombasa	1994	15–39	36	228	15·8	2·92	5·42
Kenya	1993–2017	Mombasa	1995	15–39	24	213	11·3	2·46	4·57
Kenya	1993–2017	Mombasa	1996	15–39	29	325	8·9	2·01	4·45
Kenya	1993–2017	Mombasa	1997	15–39	41	364	11·3	1·68	6·71
Kenya	1993–2017	Mombasa	1998	15–39	31	271	11·4	1·41	8·13
Kenya	1993–2017	Mombasa	1999	15–39	24	207	11·6	1·21	9·52
Kenya	1993–2017	Mombasa	2000	15–39	16	188	8·5	1·06	8·01
Kenya	1993–2017	Mombasa	2001	15–39	8	216	3·7	0·95	3·91
Kenya	1993–2017	Mombasa	2002	15–39	19	251	7·6	0·88	8·65
Kenya	1993–2017	Mombasa	2003	15–39	14	245	5·7	0·79	7·23
Kenya	1993–2017	Mombasa	2004	15–39	13	263	5·0	0·74	6·69
Kenya	1993–2017	Mombasa	2005	15–39	14	271	5·2	0·70	7·39
Kenya	1993–2017	Mombasa	2006	15–39	8	228	3·5	0·65	5·36
Kenya	1993–2017	Mombasa	2007	15–39	7	244	2·9	0·62	4·60
Kenya	1993–2017	Mombasa	2008	15–39	4	211	1·9	0·58	3·24
Kenya	1993–2017	Mombasa	2009	15–39	9	163	5·5	0·56	9·91
Kenya	1993–2017	Mombasa	2010	15–39	3	187	1·6	0·52	3·09
Kenya	1993–2017	Mombasa	2011	15–39	3	283	1·1	0·48	2·19
Kenya	1993–2017	Mombasa	2012	15–39	2	334	0·6	0·44	1·36
Kenya	1993–2017	Mombasa	2013	15–39	4	280	1·4	0·40	3·55
Kenya	1993–2017	Mombasa	2014	15–39	5	225	2·2	0·37	6·08
Kenya	1993–2017	Mombasa	2015	15–39	5	222	2·3	0·33	6·71
Kenya	1993–2017	Mombasa	2016	15–39	4	216	1·9	0·30	6·28
Kenya	1993–2017	Mombasa	2017	15–39	3	237	1·3	0·26	4·88
**McKinnon et al (2015)**^43^									
Kenya	2008–11	Nairobi	2010	15–39	34	1514	2·2 (1·6–3·1)	0·63	3·49
**Nagot et al (2005)**^44^									
Burkina Faso	1998–2002	Bobo-Dioulasso	2000	15–39	19	594	3·2 (1·9–4·9)	0·20	15·87
**Naicker et al (2015)**^45^									
South Africa	2004–06	Durban	2005	25–49	10	248	4·0 (1·9–7·4)	2·09	1·93
South Africa	2004–06	Durban	2005	15–24	8	59	13·5 (5·8–26·6)	2·93	4·62
**Nouaman et al (2022)**^46^									
Cote d'Ivoire	2016–17	San Pedro	2017	15–39	4	188	3·3	0·09	37·94
Cote d'Ivoire	2016–17	Abidjan	2017	15–39	3	293	1·6	0·17	9·60
**Price et al (2012)**^47^									
Kenya	2008	Kilifi	2007	15–39	9	339	2·7 (1·4–5·1)	0·39	6·94
Kenya	2008	Nairobi	2007	15–39	2	527	0·4 (0·1–1·5)	0·76	0·53
**Priddy et al (2011)**^48^									
Kenya	2008	Nairobi (Mukuru District)	2008	15–39	5	89	5·6 (1·6–12·0)	0·71	7·88
**Riedner et al (2006)**^49^									
Tanzania	2000–03	Mbeya Region	2002	15–39	19	99	19·2	1·47	13·08
**Roddy et al (1998)**^50^									
Cameroon	1994–96	Yaounde–Douala	1995	15–39	46	698	6·6	0·84	7·87
**Thirumurthy et al (2021)**^18^‖									
Kenya	2017–20	Siaya County	2018	15–39	0	939	0·0	0·44	0·00
**Van Damme et al (2002)**^51^									
South Africa	1996–2000	KwaZulu-Natal	1998	15–39	30	182	16·5	3·45	4·78
Benin	1996–2000	Cotonou	1998	15–39	10	121	8·3	0·90	9·21
Cote d'Ivoire	1996–2000	Abidjan	1998	15–39	5	67	7·4	1·62	4·57
**van der Loeff et al (2001)**^52^									
Guinea-Bissau	1989–98	Guinea-Bissau	1994	15–39	3	126	2·4 (0·7–7·4)	0·44	5·39
Guinea-Bissau	1989–98	Guinea-Bissau	1994	40–49	7	160	4·4 (2·1–9·2)	0·24	18·55
Guinea-Bissau	1989–98	Guinea-Bissau	1994	50–59	5	67	7·5 (3·1–18·0)	0·13	56·66

The referenced study was taken as the primary study from which estimates were extracted from, or from which unpublished estimates were received. Unpublished incidence estimates among women who engage in sex work and person-years of follow-up were obtained through personal communications with study authors. IRR=incidence rate ratio.

* Area used to match women who engage in sex work incidence to total female population incidence estimates to calculate IRRs.

† Age group used to match women who engage in sex work incidence to total female population incidence estimates; when no age information was specified, a default of age 15–39 years was used.

‡ Incidence matched to women who engage in sex work estimate by subnational location, midpoint year, and age group.

§ IRR calculated by dividing study-reported women who engage in sex work incidence by area–year matched total population female incidence. Total population female incidence in 2022 was extracted from Naomi, the UNAIDS-supported district-level estimation model,^22^ and extrapolated parallel to national-level female HIV incidence trajectories from UNAIDS Global HIV Estimates 2022, National Spectrum estimates.^23^, ^24^

¶ Incidence estimate were derived from serial cross-sectional prevalence testing with no estimate of person-years or number of new infections. The number of person-years was imputed using the median number of person-years across all studies and split proportionally according to the denominator in each age group. Imputed person-year values were then multiplied by the study-reported HIV incidence to back-calculate the number of HIV infections.

‖ Incidence estimate derived from recent HIV infection testing. The number of person-years was derived by multiplying the number of tested individuals by 0·5.

**Table 2:** Study characteristics

	**Country**	**Study population age, years**	**Definition of women who engage in sex work**	**Study design and recruitment**	**Incidence estimation method**	Additional papers identified*
Ali et al (2022)^15^	Zimbabwe	18–39	Women who self-reported exchanging sex for money in the past 30 days and had been living or working in the survey site for at least 6 months	Cross-sectional respondent-driven sampling surveys conducted between 2011 and 2017 at sex work hotspots	Prevalence back calculation, pooling data from RDS surveys; estimation of HIV incidence from analysis of HIV prevalence patterns	NA
Braunstein et al (2011)^29^	Rwanda	Median: 24, range: 18–46	Women who had exchanged sex for money at least once in the past month or were currently having sex with multiple partners plus having sex at least twice per week (all enrolled women self-identified as sex workers)	Cohort recruited through community meetings conducted by community mobilisers	Seroconversion at follow-up 6–12 months after baseline survey; midpoint estimation between last HIV-negative and first-HIV positive test	Braunstein et al (2012),^53^ Braunstein et al (2011)^54^
Botswana Ministry of Health (2013)^28^	Botswana	≥18	Women who received either money or a gift or incentive in exchange for sexual favours within the past 3 months	Time-location sampling at hotspots for recruitment to cross-sectional IBBS survey	Recent infection testing algorithm; BED incidence assay	NA
Chabata et al (2021)^30^	Zimbabwe	18–24	Young women who had exchanged sex for money or material support in the past month, and explicitly stated that sex acts with men would not have happened in the absence of an exchange, and if they were not planning to move from the site within the next 6 months	Non-randomised plausibility evaluation of DREAMS on HIV incidence; respondent-driven sampling with seeds selected from sex-work hotspots identified through a community mapping	Seroconversion at follow-up 24 months post-recruitment; midpoint estimation between an HIV-negative and HIV-positive test	NA
Chersich et al (2014)^31^	Kenya	≥16; mean 25·1 (SD 5·2)	Women reporting receipt of money in exchange for sex as part of their livelihood in the past 6 months, sexually active in the past 3 months, and not pregnant at the time of enrolment	Cohort recruited in locations with existing community links through long-standing service provision by implementers and peers through snowball sampling	Seroconversion between quarterly follow-ups; midpoint estimation between an HIV-negative and HIV-positive test	NA
Diabeté et al (2018)^32^	Benin	≥18	Women attending Dispensaire IST, the main clinic dedicated to women who engage in sex work in Cotonou	Clinic recruited cohort; all women attending invited to participate	Seroconversion between quarterly follow-ups; midpoint estimation between an HIV-negative and HIV-positive test	NA
Faini et al (2022)^33^	Tanzania	18–45	Women self-identifying as sex workers who resided within Dar es Salaam, reported to have exchanged sexual intercourse for money within the past month, considered themselves to be at increased risk for HIV infection and willing to undergo pregnancy testing	Respondent-driven sampling recruited cohort	Seroconversion follow-up visits at 3, 6, 9, and 12 months; midpoint estimation between an HIV-negative and HIV-positive test	NA
Forbi et al (2011)^34^	Nigeria	18–35	Active women who engage in sex work living in brothels within Nasarawa state of North Central Nigeria (results show all reported >1 partner per week)	Cross-sectional cohort recruited from brothels	Recent infection testing algorithm; BED assay; calculation used Hargrove adjustments^55^ and McWalter and Welte's correction^56^	NA
Fowke et al (1996)^35^	Kenya	..	Women who engage in sex work of lower socioeconomic status (from a slum area in Nairobi) who engage in sex work from their home	Cohort recruited from an existing community-based cohort established in 1985	Seroconversion between follow-ups every 6 months; midpoint estimation	NA
Ghys et al (2001)^36^	Côte d'Ivoire	Median 27, (IQR 22–32)	Women who engage in sex work attending the Clinique de Confiance, a HIV/STD clinic only available for those who are women who engage in sex work or their stable sex partners	Intervention study—peer educator recruited for a survey before recruitment of HIV-negative women who engage in sex work for the study	Seroconversion between follow-ups every 6 months (HIV negative to HIV-1 seropositive; HIV-2 seropositive to both HIV-2 and HIV-1 seropositive; HIV-negative to HIV-2 seropositive); midpoint estimation	NA
Gilbert et al (2003)^37^	Senegal	Mean 30·4 (range 19–56)	Registered sex workers (self-identifying sex workers were required by government to register and regularly attend a health clinic)	Clinic-recruited cohort	Seroconversion between follow-ups every 6 months; midpoint estimation	Travers et al (1995),^57^ Kanki et al (1994),^58^ Kanki et al (1999)^59^
Jones (2023)^20^	Zimbabwe	Median age at first test: 27	Women attending programme clinics (predominantly cisgender women who self-identify as selling sex)	Routinely collected clinic data from CeSHHAR Zimbabwe's Key Populations Programme, which encompasses a national sex worker programme including community outreach	Seroconversion between two tests after first attending the programme; midpoint estimation	Jones et al (2022),^60^ Hargreaves et al (2016)^61^
Kasamba et al (2019)^38^	Uganda	≥18 (<18 included if pregnant, had children, or provided for their own livelihood)	Women who reported engaging in commercial sex (self-identified women who engage in sex work or received money, goods, or other favours in exchange for sex) or employed in an entertainment facility; analysis only included those who reported at follow-up that their sole source of income was sex work, or sex work and another job (results excluded those who did not engage in sex work)	Peer-recruited cohort at mapped sex work hotspots	Seroconversion between 3-monthly follow-ups; random estimation with uniform distribution	Vandepitte et al (2014),^62^ Redd et al (2014),^63^ Kasamba et al (2019),^64^ Abaasa et al (2021),^65^ Abaasa et al (2019)^66^
Kassanjee et al (2022)^14^	South Africa	≥18; median 32 (IQR 27–38)	Cisgender women who had sold or transacted in sex in the past 6 months and worked in one of the districts that were studied	Cross-sectional respondent driven sampling survey recruitment at hotspots visited by outreach programmes	Recent infection testing algorithm; Kassanjee method for incidence calculation; MDRI 145, FRR 0·50%	NA
Kaul et al (2004)^39^	Kenya	≥18; mean 29·1 (SD 7·8)	Women who reported having received money or gifts in exchange for sex over the past month	RCT recruited through a series of community visits assisted by peer educators	Seroconversion between follow-ups every 6 months	Kaul et al (2004),^67^ Peterson et al (2013),^68^ Plummer et al (1991),^69^ Willerford et al (1993),^70^ MacDonald et al (2000)^71^
Kerrigan et al (2019)^40^	Tanzania	≥18; mean 27·8	Women who reported exchanging sex for money in the past month	RCT recruited through time location sampling at entertainment venues	Seroconversion between baseline and follow-up at 18 months	NA
Kilburn (2018)^25^	South Africa	Recruited ages 13–20, enrolled in high school; median 15 (IQR 14–17)	Young women who reported transactional sex (where they felt that they had to have sex with a male partner as he gave them money or gifts) with any partner in the past 12 months	RCT recruited through the Agincourt Health and Social Demographic Surveillance System; participants visited at home to check eligibility for enrolment	All participants were assessed before random assignment and then reassessed annually at 12, 24, and 36 months until they graduated from high school or the study ended	Ranganathan et al (2020)^72^
Laga et al (1994)^41^	Democratic Republic of Congo	..	Women who self-identified as sex workers	Cohort study (recruitment method not reported)	6-monthly HIV-1 incidence rates were computed assuming that seroconversion had occurred at midpoint between the first positive HIV-1 serological test and the last negative one	Laga et al (1993)^73^
Lyons et al (2020)^42^	Senegal	≥18; mean 38·5 (IQR 30–45)	Women assigned female at birth and having engaged in sex work as a primary source of income during the year before enrolment	Cohort recruited through respondent-driven sampling with additional purposive sample recruitment	Time to event survival analysis (seroconversion date was the diagnosis date); 4-month follow-up visits	NA
Malama et al (2022)^17^	Zambia	18–45	Women who reported currently exchanging sex for money	Community-recruited cohort through peers and health-care workers at bars, lodges, and on streets	First visit 1 month after enrolment, then 2 months later, then quarterly	NA
McClelland et al (2015)^19^	Kenya	≥18; median 31 (IQR 26–37)	Participants self-reported exchanging sex for cash or in-kind payment; most women reported working in bars, where they met local male clients	1993–97: clinic recruited cohort with outreach meetings in bars; 1998–2017: cohort recruited through peer-led community outreach meetings at bars	Seroconversion estimated between monthly follow-ups; for women who acquired HIV and a viral load was first detected at or after seroconversion, the date of infection was estimated at the midpoint between the last seronegative and first seropositive test; for women with a detectable viral load before seroconversion (ie, HIV RNA detected but antibodies were not), infection date was estimated as 17 days before the positive viral load	Baeten et al (2007),^74^ Baeten et al (2005),^75^ Graham et al (2014),^76^ Graham et al (2013),^77^ Lavreys et al (2000),^78^ Lavreys et al (2002)^79^ Martin et al (1998),^80^ Martin et al (2005),^81^ Martin et al (1999),^82^ McClelland et al (2006),^83^ McClelland et al (2006),^84^ McClelland et al (2005),^85^ McClelland et al (2007),^86^ McClelland et al (2018),^87^ Richardson et al (2001),^88^ Sabo et al (2019),^89^ Willcox et al (2021)^90^
McKinnon et al (2015)^43^	Kenya	..	Anyone enrolled at the SWOP-City clinic, a sex worker outreach programme offering integrated HIV prevention, care, and treatment services	Community-recruited cohort at hotspots through peers and health-care workers	Seroconversion between quarterly follow-ups	NA
Nagot et al (2005)^44^	Burkina Faso	15–56	Professional sex workers (“seaters” and “roamers”, averaging 18–28 clients per week) and non-professional sex workers (waitresses, fruit/beer sellers, students) who did not identify as a sex workers but reported an average of 2–3 clients per week	Community-recruited cohort at workplaces through peers	Seroconversion during follow-up visits which took place every 3 months	NA
Naicker et al (2015)^45^	South Africa	≥18	Self-identifying sex workers	Purposively recruited cohort through community liaison partners	Seroconversion during monthly follow-ups; midpoint estimation	van Loggerenberg et al (2008)^91^
Nouaman et al (2022)^46^	Côte d'Ivoire	≥18; median 25 (IQR 21–29)	Women who engage in sex work working at a site of prostitution at the time of the study	Cross-sectional convenience sample recruitment by CBO staff	Recent infection testing algorithm; MDRI 0·3 years, FRR 0·013	NA
Price et al (2012)^47^	Kenya	Kilifi: median 25 (range 18–65); Nairobi: median 28 (range 18–59)	Women who had received goods or money for sex	Cohort recruitment through hotspots, VCT centres, and peer recruitment	Seroconversion between quarterly follow-ups	NA
Priddy et al (2011)^48^	Kenya	Mean 28 (range 18–55)	Women aged 18–60 years who were HIV negative and not pregnant, and who reported exchanging sex for money or gifts at least three times in the past month	Cohort recruited HIV-negative women who attended education sessions for female sex workers in the Mukuru neighbourhood of Nairobi	Seroconversion at follow-up 6 months after baseline	NA
Riedner et al (2006)^49^	Tanzania	16–39	Women working in modern and traditional bars, guesthouses, and hotels	Cohort recruited from project sites (seem to be hotspots)	Seroconversion between 3-monthly follow-ups; midpoint estimation	NA
Roddy et al (1998)^50^	Cameroon	18–45; mean 26	Female sex workers residing in Yaoundé or Douala, Cameroon, who averaged at least four sexual partners per month	RCT recruitment not clear	Seroconversion at yearly follow-up	NA
Thirumurthy et al (2021)^18^	Kenya	≥18; median 25 (IQR 22–31)	Women who reported sex work as their primary or secondary source of income with ≥2 male sexual partners in the past 4 weeks	Cohort recruited through random sampling in beach and hotspot clusters from a list of all eligible women who engage in sex work	Seroconversion between 6-monhtly follow-ups	NA
Van Damme et al (2002)^51^	Benin, Côte d'Ivoire, South Africa	≥18 (≥16 South Africa)	No explicit sex work definition provided	RCT clinic recruitment	Seroconversion between follow-ups occurring every 2 months; midpoint estimation	Auvert et al (2011)^92^
van der Loeff et al (2001)^52^	Guinea Bissau	≥15; median 28·5 (IQR 21·2–43·5)	Commercial sex workers (no further definition given)	Cohort recruited from a string of villages in northwest Guinea-Bissau	Seroconversion between first survey (occurring between 1989 and 1992) and second survey (occurring between 1996 and 1998)	NA

CBO=Community-based organisation. CeSHHAR=The Centre for Sexual Health and HIV/AIDS Research Zimbabwe. DREAMS=Determined Resilient Empowered AIDS-free Mentored and Safe women. FRR=False recency rate. IBBS=Integrated bio-behavioural survey. MDRI=Mean duration of recent infection. RCT=randomised controlled trial. RDS=Respondent driven sampling. STD=sexually transmitted disease. SWOP=Sex Workers Outreach Project. VCT=Voluntary counselling and testing.

* Instances where multiple papers from the same study were identified; these papers were not used for quality assessment or incidence estimate extraction. Note that language used in this table is taken directly from the cited studies.

19 of 32 studies were cohort studies, seven were randomised control trials or intervention studies, five were cross-sectional studies, and one used routine clinic data (table 2). Seven studies included women who self-identified as sex workers, 13 included women who exchanged sex for money or goods (either over a defined period or as a primary or secondary source of income), two studies included women who worked in a known sex work location. No studies were identified on transgender women who engage in sex work or stratified by gender identity. Six study populations were women linked to clinics or sex worker programmes. Study reach varied widely, from studies in single clinics to single towns, cities, or regions, and multiple locations nationally in South Africa and Zimbabwe. Recruitment methods included network sampling approaches (seven studies), time location sampling (four), clinic recruitment (seven), or convenience samples from peer outreach activities or community meetings (nine). Study participant ages ranged from a median of 15 years (IQR 14–17)^27^ to a mean of 38 years (30–45),^93^ with 11 (34%) studies reporting mean or median ages between 25 and 30 years. Incidence estimates were predominantly derived from inference of a seroconversion date between HIV tests (27 studies). Most of these studies used the midpoint between first positive test result and last negative test result (14 [52%] of 27), two used the HIV-positive test date, and ten did not report a clear seroconversion date estimation approach. Four studies estimated incidence from recency assays, and one used back calculation from age-specific HIV prevalence.

Assessment with the GHQAT classified 17 studies as good quality and the remaining 15 as fair (appendix 2 p 10). Studies all scored well in terms of study design, with study objectives and study populations clearly defined. 26 studies either had robust approaches to sampling or did not seek to generalise their findings beyond their study population so were considered adequately representative. Power calculations were presented in nine studies. Scores for study implementation varied, with ten studies reporting participation rates, of which six reported over 85% participation. For 19 studies, it was likely that participants enrolled were representative of the source population. Scoring for measurement of HIV incidence was variable. Among longitudinal studies, 12 report over 70% participant retention at 12 months or study endpoint and 15 assessed reasons for dropout or described methods to address loss to follow-up.

**Figure 2:**
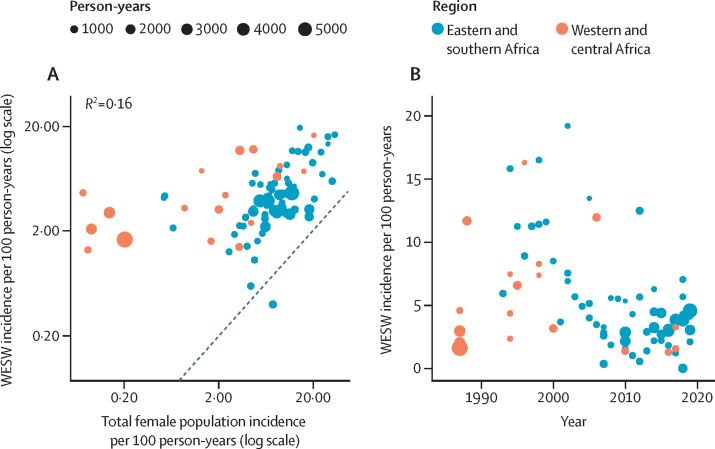
Empirical estimates of HIV Incidence among women who engage in sex work Association between HIV incidence in WESW and in the district-year-sex matched total population (A) and empirical estimates of HIV incidence in WESW over time (B). Black dashed line represents the line of equality. WESW=women who engage in sex work.

**Figure 3:**
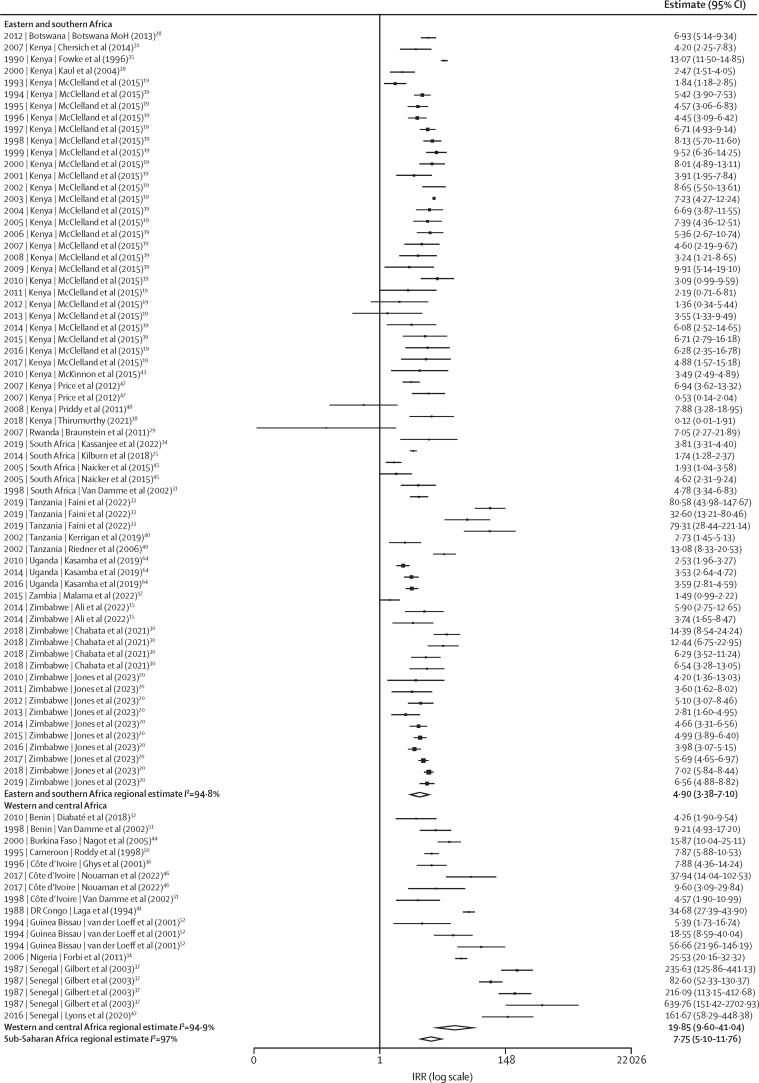
Meta-analysis of HIV incidence in women who engage in sex work relative to the total female population in sub-Saharan Africa IRRs calculated by dividing empirical estimates of HIV incidence in women who engage in sex work by HIV incidence among total population of women matched for age, district, and year derived from the district-level estimation model Naomi,^22^ and synthesised by use of meta-analysis with study-district random effects. Figure shows year of data collection, country, and study.

Incidence among women who engage in sex work was correlated with matched female incidence (Pearson's correlation coefficient=0·6 [95% CI 0·5–0·8]; p<0·0001; figure 2). In meta-analysis, HIV incidence among women who engage in sex work was almost eight times higher than in matched total population women (IRR 7·8 [95% CI 5·1–11·8]; figure 3). IRRs were greater in western and central Africa (19·9 [9·6–41·0]) than in eastern and southern Africa (4·9 [3·4–7·1]). Heterogeneity across IRR estimates was high (sub-Saharan Africa *I*^2^ 96·9%, eastern and southern Africa *I*^2^ 94·7%, western and central Africa *I*^2^ 94·9%; figure 1), although median IRRs were similar to pooled IRRs from meta-analysis (sub-Saharan Africa median IRR: 6·1, IQR 3·9–9·4; appendix 2 p 12). Sensitivity analysis using the 20 studies scoring above 60% in the quality assessment yielded a pooled IRR of 7·0 (95% CI 4·1–12·0; appendix 2 p 7). Sensitivity analysis using nationally matched incidence resulted in higher IRR (9·5 [95% CI 6·6–13·7]; appendix 2 p 8).

**Figure 4:**
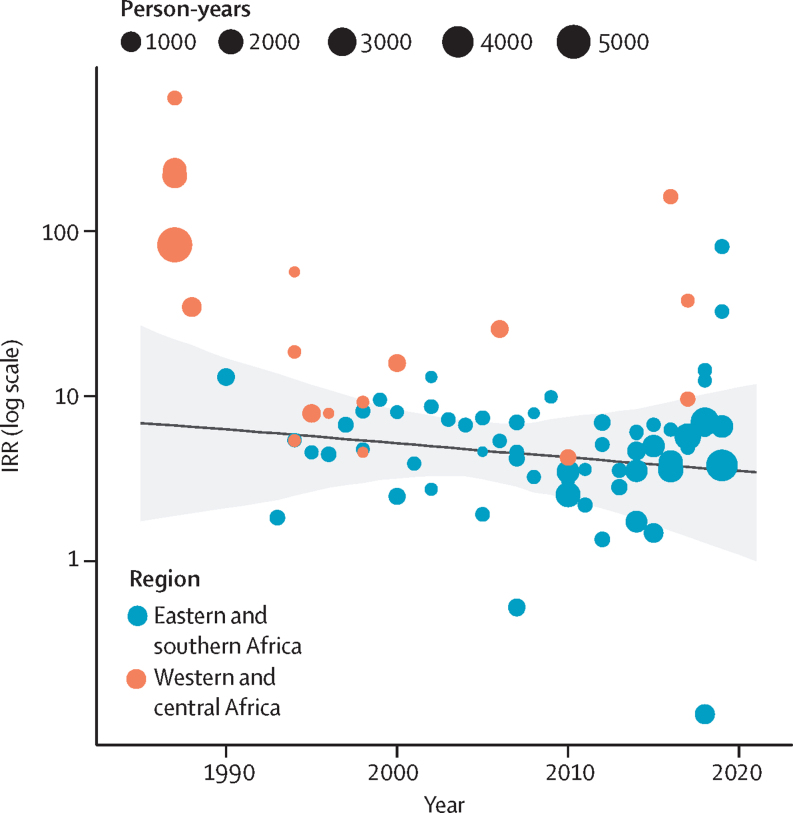
HIV IRRs modelled over time, presented on the logarithmic scale Points represent IRRs calculated by dividing study-reported HIV incidence in WESW by age–district–year matched total population HIV incidence derived from the Naomi model.^22^ The solid line represents the estimated IRR for sub-Saharan Africa, with the grey shading capturing the 95% uncertainty range. IRR=incidence rate ratio. WESW=women who engage in sex work.

There was no evidence for a change in IRR over time (IRR per 5 years 0·9 [95% CI 0·7–1·2]; figure 4). Incidence in women who engage in sex work was nearly five times higher than in the total female population in 2003 estimated from the log-linear mixed-effects model (IRR 4·9 [95% CI 3·3–7·3]; figure 3; appendix 2 p 13).

In separate models fitted to data from Kenya and Zimbabwe, log IRR did not change over time in Kenya (IRR per 5 years: 1·0 [95% CI 1·0–1·1]) but increased in Zimbabwe between 2009 and 2019 (1·6 [1·3–2·1]; appendix 2 p 12). Two studies from these countries provided annual incidence estimates (appendix 2 p 9). In the Kenyan cohort, incidence among women who engage in sex work peaked at 15·8 per 100 person years in 1994 and declined to 1·3 per 100 person years in 2017. However, the declines in incidence among women who engage in sex work were matched by reductions in total female population incidence, resulting in a stable IRR over the cohort period. In the Zimbabwean cohort, the HIV incidence rate in women who engage in sex work also declined from 5·4 per 100 person years to 3·1 per 100 person years from 2010 to 2019, but the IRR increased due to faster relative declines in total population female incidence.

## Discussion

In sub-Saharan Africa, HIV incidence among women who engage in sex work was nearly eight times higher than in the total female population; this disparity was larger in western and central Africa, where incidence was almost 20 times higher, compared with five times higher in eastern and southern Africa. Between 1985 and 2020, incidence in women who engage in sex work declined at a similar rate to incidence in matched women in the total population; however, case studies of Kenya and Zimbabwe illustrated that these trends might vary between countries. Sensitivity analyses using national incidence estimates and restricted to high-quality studies did not alter the interpretation of our findings.

Our analysis adds to existing evidence that women who engage in sex work are disproportionately affected by HIV. High relative incidence echoes the five-fold higher HIV prevalence among women who engage in sex work compared with all adult women in sub-Saharan Africa.^2^ Relative incidence was higher in western and central Africa, where overall HIV prevalence and incidence are lower than eastern and southern Africa. These findings mirror similar findings of a larger HIV prevalence gap between women who engage in sex work and the total female population in western and central Africa than in eastern and southern Africa.^2^ The regional differential in IRRs primarily reflects differences in population HIV incidence (the denominator for the IRR) and transmission dynamics between the regions, rather than differential incidence among women who engage in sex work.^93^ Despite higher relative incidence in western and central Africa, median incidence was modestly lower than in studies in eastern and southern Africa. HIV transmission in western and central Africa tends to be more concentrated among key populations such as women who engage in sex work, resulting in larger incidence differences with the total population.^94^

The declining HIV incidence among women who engage in sex work observed in this analysis was consistent with mathematical modelling studies from western and central Africa that estimate the proportion of total new HIV infections attributable to commercial sex has fallen over time.^95^, ^96^ These parallel declines contrast HIV incidence trends among men who have sex with men in sub-Saharan Africa for whom a recent systematic review found no evidence of incidence decline.^97^ Initial reductions in incidence in women who engage in sex work probably resulted from implementation of sex worker dedicated prevention programmes and health services in sub-Saharan Africa.^5^ More recently, declines might reflect the effect of HIV treatment as prevention, including increasing treatment coverage among men.^1^

Studies identified in this review varied in their geographical scope, recruitment, and definition of women who engage in sex work. Inclusion criteria often defined the age range and duration or frequency of selling sex, or enrolled women self-identifying as women who engage in sex work, leading to a range of study populations with heterogeneous HIV acquisition risk. This variation is reflected by the heterogeneity in our findings. Studies among women linked to sex worker focused programmes or clinics might have recruited women at lower risk of HIV acquisition, who might be older or have sold sex for longer, potentially missing younger women, those not identifying as selling sex, and those in the highest risk period immediately following sex work initiation.^98^ Data from South Africa suggest that sex work dynamics are changing, with the mean age of sex workers and durations at-risk both increasing,^99^ while young women who engage in sex work remain at highest incidence risk.^100^ Different sampling and recruitment approaches are also likely to have identified different women for study inclusion. Studies have shown that participants recruited through respondent-driven sampling are younger than those recruited through venue-based snowball sampling.^101^ Using IRRs matched for district and age in our analysis probably mitigated some of the bias that would have come from geographical and age differences if raw estimates of incidence were analysed independently.

Our study has limitations. Data were available from less than half of sub-Saharan Africa countries, with western and central Africa particularly under-represented, and estimates for eastern and southern Africa disproportionately from Kenya and Zimbabwe. This limitation precluded estimation of country-specific IRRs and non-linear time trends and limited the generalisability of our findings. Despite the heterogeneity reported, data were insufficient to disaggregate the meta-analysis beyond region to explore factors that might have contributed to this variation; for example, age-disaggregated IRRs could have provided further insights, given evidence of higher incidence among younger women who engage in sex work.^100^ For all but one study that provided a comparator group of women not engaging in sex work,^25^ our IRR denominators were based on extrapolations of subnational estimates backwards in time parallel to national female incidence trajectories. Although this approach aimed to capture spatial variation over time, there is substantial uncertainty in these subnational incidence denominators, particularly for older studies, which is not reflected in the calculated IRRs. Additionally, given high mobility among women who engage in sex work,^11^ matching on district boundaries might not yield the most comparable total female population denominator. However, sensitivity analysis with nationally matched IRRs gave similar results to the district-matched analysis, alleviating concerns around subnational uncertainties.

Most studies did not make inferences beyond the study population they recruited and scored positively in the quality assessment on adequately representing the wider population of women who engage in sex work. However, it is unlikely that many studies would have been representative had they made these inferences. Ascertaining the degree of bias associated with sample recruitment posed challenges. There is no standardised approach to sampling individuals from key populations, and although respondent-driven sampling is widely accepted as a gold standard to achieving a representative sample,^102^ there was variation in implementation and reporting across studies.

Our review highlights that while HIV incidence data are available for women who engage in sex work, geographical gaps remain, and temporal trends are difficult to ascertain from empirical estimates alone, particularly at country level. Individual studies were challenging to compare due to large variation in study design and the limited generalisability of findings beyond individual study populations. Standardisation in definitions for women who engage in sex work and reporting of age ranges could improve comparability of estimates. Applying consistent methodological standards for measuring incidence in key populations would improve comparability, generalisability, and ability to estimate trends. Data gaps could be addressed by incorporating incidence measurements into survey and routine programmatic data analysis via serial cross-sectional prevalence data from biobehavioural surveys and recency testing.^14^, ^15^, ^20^, ^28^ Increasing dissemination and use of these data where they are already collected could facilitate more robust country-level estimation and support real-time data-driven programming.

The declining HIV incidence among women who engage in sex work in sub-Saharan Africa parallel to incidence trends among all adult women represents progress, but the persistently large disparity in HIV incidence highlights the need to sustain and continue to expand effective HIV prevention options and support for women who engage in sex work. Preventing new HIV infections though focused programmes for key populations, including options and preferences for different prevention technologies, will become increasingly important as incidence further declines in the general population.^103^ Counterfactual-based modelling shows that fully meeting HIV prevention and treatment needs of women who engage in sex work would substantially affect the HIV epidemic in sub-Saharan Africa, and that averting HIV transmission associated with sex work should remain a programmatic priority to achieve, and sustain, epidemic control.^6^, ^8^, ^96^ Transitioning from continent-level summaries to location-specific and programme-specific monitoring to ensure inequalities are identified and addressed in a timely and efficient manner will require expanded future surveillance activities with more standardised approaches to obtaining empirical estimates and repeat measures in the same populations.

### Contributors

### Data sharing

Data collected for this study did not include individual participant data. All aggregate data extracted from published literature or provided by co-authors and analysed in meta-analysis are reported in table 1. Data in spreadsheet format and R code to reproduce analysis are available from: https://github.com/rebecca-and/WESW-incidence.git.

## Declaration of interests

We declare no competing interests.

## Supplementary Materials

French translation of the abstract
