# Supplementary material for: HIV incidence among women engaging in sex work in sub-Saharan Africa: a systematic review and meta-analysis
Source: Lancet Glob Health. 2024 Jul 17;12(8):e1244–60. doi: 10.1016/S2214-109X(24)00227-4 (PMC11283888; doi:10.1016/S2214-109X(24)00227-4)
Supplement: French translation of the abstract [file mmc1.pdf]

THE LANCET
Global Health

Supplementary appendix 1

This translation in French was submitted by the authors and we reproduce it as supplied. It has not been peer reviewed. *The Lancet*'s editorial processes have only been applied to the original in English, which should serve as reference for this manuscript.

Cette traduction en français a été proposée par les auteurs et nous l'avons reproduite telle quelle. Elle n'a pas été examinée par des pairs. Les processus éditoriaux du *Lancet* n'ont été appliqués qu'à l'original en anglais et c'est cette version qui doit servir de référence pour ce manuscrit.

Supplement to: Jones HS, Anderson RL, Cust H, et al. HIV incidence among women engaging in sex work in sub-Saharan Africa: a systematic review and meta-analysis. *Lancet Glob Health* 2024; **12:** e1244–60.

Résumé

Contexte

Des travailleuses du sexe (TS) en Afrique subsaharienne ont un risque élevé d'infection par le VIH. L'incidence du VIH chez des femmes en Afrique subsaharienne a diminué, mais il existe une faible connaissance des tendances de l'incidence chez des TS. Nous avons examiné l'incidence du VIH chez les TS, comparativement à la population féminine pour comprendre les disparités dans l'incidence et des tendances au fil du temps.

Méthodes

On a effectué une recherche dans les bases de données Medline, Embase, Global Health et Google Scholar de janvier 1990 à février 2024 et la littérature grise pour trouver des estimations empiriques de l'incidence du VIH chez les TS en Afrique subsaharienne. Pour chaque estimation, nous avons calculé un « Incidence Rate Ratio » (IRR) (le ratio entre l'incidence chez les TS, et l'incidence chez la population féminine, appariée selon l'âge, l'année et la zone. On a mené une métaanalyse utilisant les IRRs. Nous avons modélisé les changements dans les IRRs au fil du temps avec un modèle mixte.

Résultats

Il y avait 32 études menées entre 1985 et 2020 avec 2 194 nouvelles infections à VIH chez les TS pour 51 490 personnes-années (pa). L'incidence médiane chez les TS était de 4,3/100 py (intervalle interquartile : 2,8-7,0/100 py). L'incidence chez les WESW était huit fois plus élevée que chez les femmes appariées (IRR 7,8, IC à 95 % : 5,1-11,8), avec une différence relative plus grande en Afrique occidentale et centrale (IRR 19,9, IC à 95 % : 9,6-41,0) qu'en Afrique orientale et australe (IRR 4,9, IC à 95 % : 3,4–7,1). Il n'y avait aucune preuve d'un changement de l'IRR avec le temps (IRR par 5 années : 0,9, 95% CI: 0,6,1,3%).

Conclusions

En Afrique subsaharienne, l'incidence du VIH chez les TS reste disproportionnellement élevée par rapport à la population féminine, cependant, l'incidence relative est constante depuis des années indique que l'incidence chez les TS et l'incidence chez la population féminine ont reculé dans une mesure équivalente. Les estimations de l'incidence par zone sont rares. Une surveillance améliorée et la standardisation des approches de mesure de l'incidence pourraient améliorer la disponibilité des données. Pour réduire les inégalités persistantes, il est essentiel que les efforts de prévention du VIH soient consolidés et qu'ils se poursuivent.

Financement

Bill & Melinda Gates Foundation, UKRI, NIH
