# Supplementary material for: HIV incidence among women engaging in sex work in sub-Saharan Africa: a systematic review and meta-analysis
Source: Lancet Glob Health. 2024 Jul 17;12(8):e1244–60. doi: 10.1016/S2214-109X(24)00227-4 (PMC11283888; doi:10.1016/S2214-109X(24)00227-4)
Supplement: Supplementary appendix 2 [file mmc2.pdf]

THE LANCET
Global Health

Supplementary appendix 2

This appendix formed part of the original submission and has been peer reviewed. We post it as supplied by the authors.

Supplement to: Jones HS, Anderson RL, Cust H, et al. HIV incidence among women engaging in sex work in sub-Saharan Africa: a systematic review and meta-analysis. *Lancet Glob Health* 2024; **12:** e1244–60.

Table of Contents

Supplementary Text S1: Search strategies for the reviews. 2

Supplementary Text S2: Bayesian log-linear mixed-effects model description 5

Supplementary Figure S1: Meta-analysis of HIV incidence in women who engage in sex work relative to the total female population in Sub-Saharan Africa using only high-quality studies. 6

Supplementary Figure S3: Case Studies - Incidence and Incidence Rate Ratio (IRR) trends over time in data from Mombasa, Kenya (McClelland et al) and Zimbabwe (Jones et al). 8

Supplementary Table S1: Global HIV Quality Assessment Tool Summary Table 9

Supplementary Table S2: Median incidence rate ratios for in women who engage in sex work relative to the national-matched total female population in Eastern and Southern Africa, Western and Central Africa, and Sub-Saharan Africa 11

Supplementary Table S3: Regression model summaries 11

Supplementary Text S1: Search strategies for the reviews.

Initial search: 19 June 2019; Date restrictions applied: 1 January 1990 – 19 June 2019

**MEDLINE Searches (Ovid MEDLINE(R) and Epub Ahead of Print, In-Process & Other Non-Indexed Citations, Daily and Versions(R) 1946 to June 04, 2019) (saved)**		
**Sex Workers**	**HIV**	**sub-Saharan Africa**
1. Sex Work/	1. exp HIV/	1. africa/ or exp "africa south of the sahara"/
2. Sex Workers/	2. Acquired Immunodeficiency Syndrome/	2. Africa.mp.
3. (sex adj2 work*).mp.	3. exp HIV Seroprevalence/	3. (((Angola or Benin or Botswana or Burkina Faso or Burundi or Cabo Verde or Cameroon or Central African Republic or Chad or Comoros or Dem* Rep* Congo or Rep* Congo or Cote D'ivoire or Equatorial Guinea or Eritrea or Eswatini or Ethiopia or Gabon or Gambia or Ghana or Guinea or Guinea-Bissau or Kenya or Lesotho or Liberia or Madagascar or Malawi or Mali or Mauritania or Mauritius or Mozambique or Namibia or Niger or Nigeria or Rwanda or Sao Tome) and Principe) or Senegal or Seychelles or Sierra Leone or Somalia or South Africa or South Sudan or Sudan or Tanzania or Togo or Uganda or Zambia or Zimbabwe).mp.
4. FSW*.mp.	4. exp HIV Seropositivity/ or exp HIV Infections/	
5. prostitut*.mp.	5. HIV.mp.	
6. transactional sex.mp.	6. human immun* virus.mp.	
7. commercial sex.mp.	7. AIDS.mp.	
8. sell* sex.mp.	8. acquire* immun* syndrome.mp.	
9. (paid adj3 sex).mp.		
10. sex industry.mp.		
11. key population*.mp.		
12. (high risk adj2 women).mp.		
13. (high risk adj2 girls).mp.		
[mp=title, abstract, original title, name of substance word, subject heading word, floating sub-heading word, keyword heading word, organism supplementary concept word, protocol supplementary concept word, rare disease supplementary concept word, unique identifier, synonyms]		

**EMBASE via Ovid**		
**Sex Workers**	**HIV**	**sub-Saharan Africa**
1. prostitution/	1. Human immunodeficiency virus/	1. Africa/
2. sex worker/	2. acquired immune deficiency syndrome/	2. exp "Africa south of the Sahara"/
3. (sex adj2 work*).mp.	3. HIV.mp.	3. Africa.mp.
4. FSW*.mp.	4. human immun* virus.mp.	4. (((Angola or Benin or Botswana or Burkina Faso or Burundi or Cabo Verde or Cameroon or Central African Republic or Chad or Comoros or Dem* Rep* Congo or Rep* Congo or Cote D'ivoire or Equatorial Guinea or Eritrea or Eswatini or Ethiopia or Gabon or Gambia or Ghana or Guinea or Guinea-Bissau or Kenya or Lesotho or Liberia or Madagascar or Malawi or Mali or Mauritania or Mauritius or Mozambique or Namibia or Niger or Nigeria or Rwanda or Sao Tome) and Principe) or Senegal or Seychelles or Sierra Leone or Somalia or South Africa or South Sudan or Sudan or Tanzania or Togo or Uganda or Zambia or Zimbabwe).mp. [mp=title, abstract, heading word, drug trade name, original title, device manufacturer, drug manufacturer, device trade name, keyword, floating subheading word, candidate term word]
5. prostitut*.mp.	5. AIDS.mp.	
6. transactional sex.mp.	6. acquire* immun* syndrome.mp.	
7. commercial sex.mp.		
8. sell* sex.mp.		
9. (paid adj3 sex).mp.		
10. sex industry.mp.		
11. key population*.mp.		
12. (high risk adj2 women).mp.		
13. (high risk adj2 girls).mp.		

**Web of Science**

Sex Workers	HIV	sub-Saharan Africa
(TS= (girls at high risk))	(TS=(human immun* virus))	(TS=Africa) AND LANGUAGE: (English)
(TS= (high risk girls))	(TS=(acquire* immun* deficiency syndrome))	
(TS= (women at high risk))	(TS=AIDS)	
(TS= (high risk women))	(TS=HIV)	
(TS=(key population*))	(TS=acquire* immun* deficiency syndrome)	
(TS= (sex industry))	(TS=human immun* virus)	
(TS= (paid for sex))		
(TS= (sell* sex))		
(TS=(commercial sex))		
(TS= (transactional sex))		
(TS=prostitut*)		
(TS=FSW*)		
(TS= (sex work*))		
Indexes=SCI-EXPANDED, SSCI, A&HCI, CPCI-S, CPCI-SSH, ESCI Timespan=1990-2019 DOCUMENT TYPES: (Article)		

Global Health via Ovid		
**Sex Workers**	**HIV**	**sub-Saharan Africa**
1. exp prostitutes/ or prostitution/	1. human immunodeficiency viruses/ or hiv infections/ or human immunodeficiency virus 1/ or human immunodeficiency virus 2/	1. africa/ or exp "africa south of sahara"/
2. exp sex workers/	2. acquired immune deficiency syndrome/	2. Africa.mp. [mp=abstract, title, original title, broad terms, heading words, identifiers, cabicodes]
3. (sex adj2 work*).mp.	3. HIV.mp.	3. (((Angola or Benin or Botswana or Burkina Faso or Burundi or Cabo Verde or Cameroon or Central African Republic or Chad or Comoros or Dem* Rep* Congo or Rep* Congo or Cote D'ivoire or Equatorial Guinea or Eritrea or Eswatini or Ethiopia or Gabon or Gambia or Ghana or Guinea or Guinea-Bissau or Kenya or Lesotho or Liberia or Madagascar or Malawi or Mali or Mauritania or Mauritius or Mozambique or Namibia or Niger or Nigeria or Rwanda or Sao Tome) and Principe) or Senegal or Seychelles or Sierra Leone or Somalia or South Africa or South Sudan or Sudan or Tanzania or Togo or Uganda or Zambia or Zimbabwe).mp. [mp=abstract, title, original title, broad terms, heading words, identifiers, cabicodes]
4. FSW*.mp.	4. human immun* virus.mp.	
5. prostitut*.mp.	5. AIDS.mp.	
6. transactional sex.mp.	6. acquire* immun* syndrome.mp.	
7. commercial sex.mp.		
8. sell* sex.mp.		
9. (paid adj3 sex).mp.		
10. sex industry.mp.		
11. key population*.mp.		
12. (high risk adj2 women).mp.		
13. (high risk adj2 girls).mp.		

Updated search: 28 February 2024; Date restrictions applied: 1 January 1990 – 28 February 2024; No language restrictions.

Medline:

1. (Kigali or Soweto or Johannesburg or Durban or Port Elizabeth or Mpumalanga or Abidjan or Harare or Mombasa or Nairobi or Brazzaville or “Dar es Salaam” or “Cape Town” or “Addis Ababa” or Douala or Accra or Luanda or Lusaka or Conakry or Kampala or Maputo or Freetown or Bangui or Bosaso).mp. [mp=abstract, title, original title, heading words, cabicodes words]
2. (Angola or Botswana or Eswatini or Swaziland or Ethiopia or Kenya or Lesotho).mp. [mp=abstract, title, original title, heading words, cabicodes words]
3. Malawi.mp.
4. (Mozambique or Namibia).mp. [mp=abstract, title, original title, heading words, cabicodes words]
5. “South Africa”.mp.
6. (“South Sudan” or Uganda or Tanzania).mp. [mp=abstract, title, original title, heading words, cabicodes words]
7. (Zambia or Zimbabwe or Benin or “Burkina Faso” or Burundi or Cameroon or “Central African Republic” or Chad or Congo or “Cote d’Ivoire” or “Ivory Coast” or “Democratic Republic of the Congo” or “Equatorial Guinea” or Gabon or Gambia or Ghana or Guinea or “Guinea-Bissau” or Liberia or Mali or Niger or Nigeria or Senegal or “Sierra Leone” or Togo).mp. [mp=abstract, title, original title, heading words, cabicodes words]
8. “Africa South of the Sahara”/

9. 1 or 2 or 3 or 4 or 5 or 6 or 7 or 8
10. exp HIV/
11. (HIV or “Human Immunodeficiency Virus” or “HIV-1”).mp. [mp=abstract, title, original title, heading words, cabicodes words]
12. (incidence or prospective or cohort or longitudinal or panel or 4rostitute4* or sero-conver*).mp. [mp=abstract, title, original title, heading words, cabicodes words]
13. (“female sex workers” or “female sex worker” or “women who sell sex” or “woman who sells sex” or prostitute* or “sex-worker” or “sex work” or “sex-work” or FSW).mp. [mp=abstract, title, original title, heading words, cabicodes words]
14. 10 or 11 or 12
15. 9 and 13 and 14
16. limit 15 to yr=”1990 – 2024”

Global Health:

1. ((Kigali or Soweto or Johannesburg or Durban or Port Elizabeth or Mpumalanga or Abidjan or Harare or Mombasa or Nairobi or Brazzaville or "Dar es Salaam" or "Cape Town" or "Addis Ababa" or Douala or Accra or Luanda or Lusaka or Conakry or Kampala or Maputo or Freetown or Bangui or Bosaso or Angola or Botswana or Eswatini or Swaziland or Ethiopia or Kenya or Lesotho or Malawi or Mozambique or Namibia or "South Africa" or "South Sudan" or Uganda or Tanzania or Zambia or Zimbabwe or Benin or "Burkina Faso" or Burundi or Cameroon or "Central African Republic" or Chad or Congo or "Cote d'Ivoire" or "Ivory Coast" or "Democratic Republic of the Congo" or "Equatorial Guinea" or Gabon or Gambia or Ghana or Guinea or "Guinea-Bissau" or Liberia or Mali or Niger or Nigeria or Senegal or "Sierra Leone" or Togo or "sub-Saharan Africa") and (HIV or "Human Immunodeficiency Virus" or "HIV-1") and (incidence or prospective or cohort or longitudinal or panel or seroconver* or sero-conver*) and ("female sex workers" or "female sex worker" or "women who sell sex" or "woman who sells sex" or prostitut* or "sex-worker" or "sex work" or "sex-work" or FSW))
2. limit 1 to yr="1990 - 2022"

EMBASE:

1. ((Kigali or Soweto or Johannesburg or Durban or Port Elizabeth or Mpumalanga or Abidjan or Harare or Mombasa or Nairobi or Brazzaville or "Dar es Salaam" or "Cape Town" or "Addis Ababa" or Douala or Accra or Luanda or Lusaka or Conakry or Kampala or Maputo or Freetown or Bangui or Bosaso or Angola or Botswana or Eswatini or Swaziland or Ethiopia or Kenya or Lesotho or Malawi or Mozambique or Namibia or "South Africa" or "South Sudan" or Uganda or Tanzania or Zambia or Zimbabwe or Benin or "Burkina Faso" or Burundi or Cameroon or "Central African Republic" or Chad or Congo or "Cote d'Ivoire" or "Ivory Coast" or "Democratic Republic of the Congo" or "Equatorial Guinea" or Gabon or Gambia or Ghana or Guinea or "Guinea-Bissau" or Liberia or Mali or Niger or Nigeria or Senegal or "Sierra Leone" or Togo or "sub-Saharan Africa") and (HIV or "Human Immunodeficiency Virus" or "HIV-1") and (incidence or prospective or cohort or longitudinal or panel or seroconver* or sero-conver*) and ("female sex workers" or "female sex worker" or "women who sell sex" or "woman who sells sex" or prostitut* or "sex-worker" or "sex work" or "sex-work" or FSW))
2. limit 1 to yr="1990 - 2022"

Google Scholar: [first five pages of results screened]

"HIV incidence" in "female sex worker*" in sub-saharan Africa

Searches above repeated using the following French terms to extend scope:

1. Kigali or Soweto or Johannesburg or Durban or Port Elizabeth or Mpumalanga or Abidjan or Harare or Mombasa or Nairobi or Brazzaville or "Dar es Salaam" or "Cape Town" or "Addis Ababa" or Douala or Accra or Luanda or Lusaka or Conakry or Kampala or Maputo or Freetown or Bangui or Bosaso or Angola or Botswana or Eswatini or Swaziland or ?thiopie or Kenya or Lesotho or Malawi or Mozambique or Namibie or "Afrique du Sud" or Sud-Soudan or “Soudan du Sud” or Ouganda or Tanzanie or Zambie or Zimbabwe or B?nin or "Burkina Faso" or Burundi or Cameroun or "R?publique centrafricaine" or "République Centre Africaine" or Tchad or Congo or "Cote d'Ivoire" or "Guin?e ?quatoriale" or Gabon or Gambie or Ghana or Guin?e or "Guin?e-Bissau" or Lib?ria or Mali or Niger or Nig?ria or S?n?gal or "Sierra Leone" or Togo or "Afrique subsaharienne"
2. VIH or sida or "virus d'immunodéficience humaine"
3. Incidence or longitudinale or cohorte or s?roconversion
4. "travailleuse* d* sexe" or "professionnelle* d* sexe" or prostitu*
5. 2 or 3
6. 1 and 4 and 5
7. **limit 6 to yr=”1990 – 2024”**

Supplementary Text S2: Bayesian log-linear mixed-effects model description

We modelled incidence rate ratios using a Bayesian mixed-effects regression model. The number of new HIV infections Y_{it} observed in women engaging in sex work (WESW) in year t in study i followed a poisson distribution:

$$Y_{it} \sim Poisson(E[y_{it}])$$

Where Y_{it} is observed new HIV infections in WESW and $E[y_{it}]$ is the expected number of infection events, which can be expressed as:

$$E[y_{it}] = \lambda_{it} * \mathrm{X}_{it}$$

Whereλ_{it} is the incidence rate in WESW and X_{it} is the person years of follow-up. We expressed the incidence rate among WESW as the product of the matched total population female HIV incidence rate and an incidence rate ratio for WESW as follows:

$$\lambda_{it} = IRR_{it} * Z_{it}$$

such that, IRR_{it} is the incidence rate ratio and Z_{it} is the district-year-sex matched total population incidence. We model $\log(IRR)$ as:

$$\log(IRR) = \beta_0 + \beta_1 t + a_s + \mathrm{b}_s t$$

$$a_s \sim N(0, \sigma_a)$$

$$b_s \sim N(0, \sigma_b)$$

whereby $\beta_0$is the intercept, $\beta_1 t$ is a fixed effect for median-centred year, a_s represents study-level random intercepts, and $\mathrm{b}_s t$ captures study-level random slopes over time. Total population incidence and person-years of follow-up are used as model offsets.

$$\log \frac{1}{\sigma_a^2}, \log \frac{1}{\sigma_b^2} \sim N(1.6, 2)$$

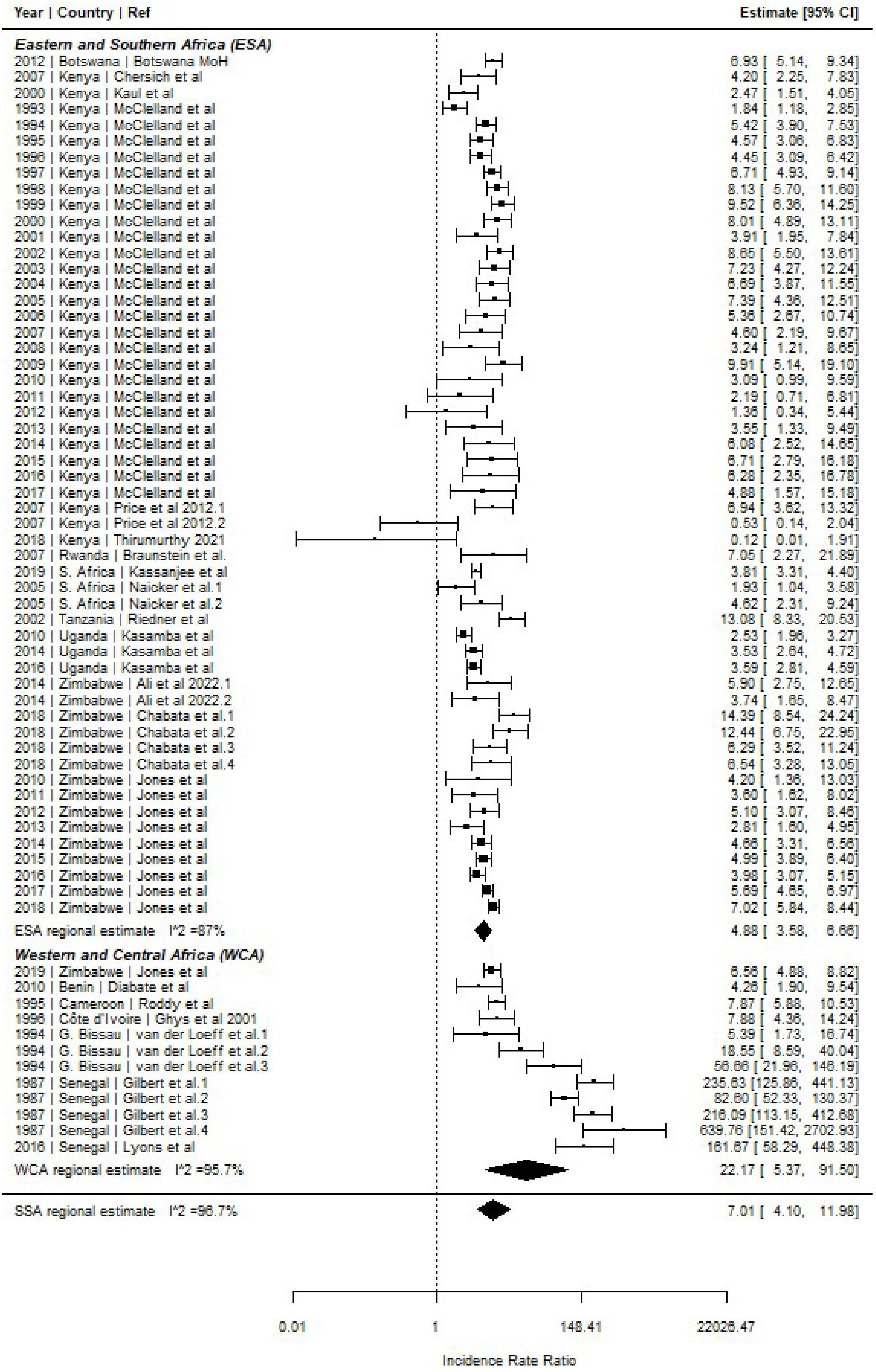

Supplementary Figure S1: Meta-analysis of HIV incidence in women who engage in sex work relative to the total female population in Sub-Saharan Africa using only high-quality studies.

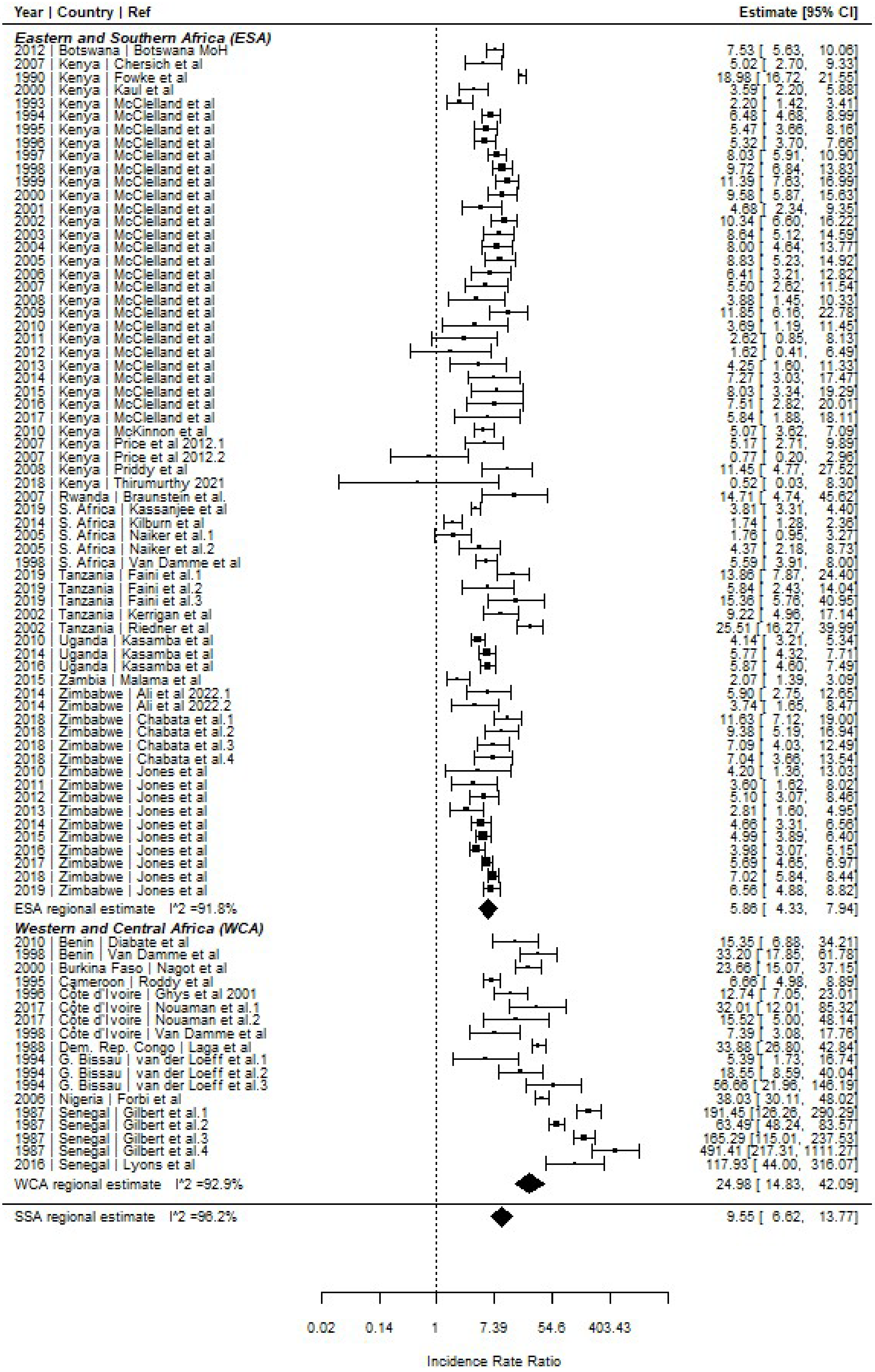

Supplementary Figure S2: Meta-analysis of HIV incidence in women who engage in sex work relative to the national-matched total female population in Sub-Saharan Africa.

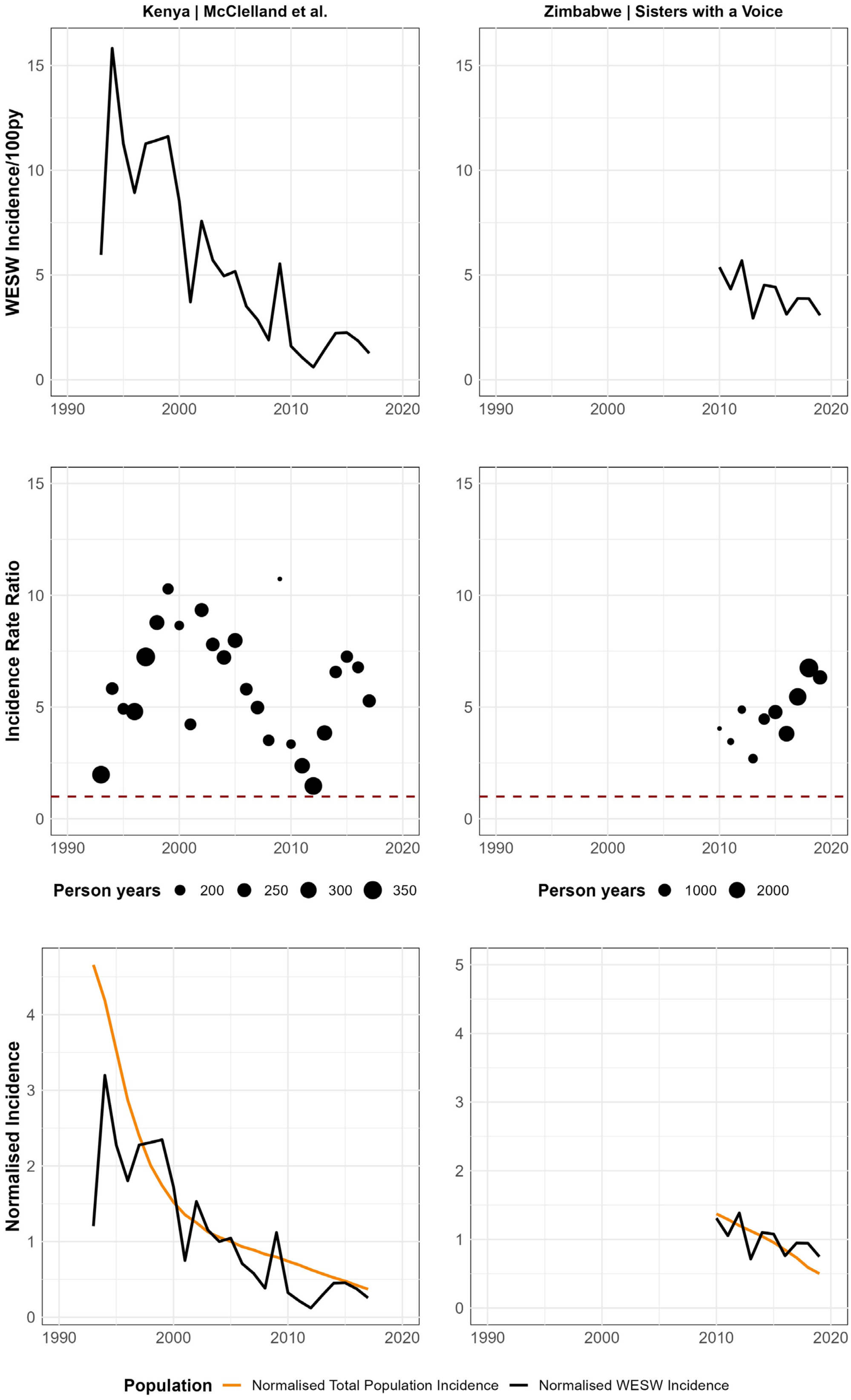

Supplementary Figure S3: Case Studies - Incidence and Incidence Rate Ratio (IRR) trends over time in data from Mombasa, Kenya (McClelland et al) and Zimbabwe (Jones et al). Upper row: Empirical incidence in women who engage in sex work (WESW) over time. Middle row: Incidence rate ratio (IRR) over time. The red dashed line represents an IRR of 1 (HIV incidence in WESW = total female population HIV incidence). Lower row: Normalised incidence over time. Calculated as a ratio of annual HIV incidence divided by incidence in the median year (1993-2018 for McClelland et al: median year = 2005; 2010-2019 for Jones et al: median year = 2015). WESW: Women who engage in sex work; IRR: Incidence Rate Ratio.

Supplementary Table S1: Global HIV Quality Assessment Tool Summary Table

	Was the research question or objective in this study clearly stated?	Was the study population clearly defined?	Will the study population defined answer the research question proposed?	Was the sample size justified either through a power description, or variance and effect estimates?	Was the proportion of people who agreed to participate reported?	Was the proportion who agreed to participate at least 85%?	Is there reason to believe that the participants enrolled are a representative sample of the source population?	Are the numerator (number newly infected with HIV) and total amount of person-time at risk reported?	Was the HIV outcome clearly defined? [a]	Were the statistical methods used to assess HIV outcome appropriate?	Have actual confidence intervals been reported?	Was the time frame sufficient so that one could reasonably expect to incident infections representative of the underlying incidence? [b]	By the end of the study, was retention reasonable? [c]	7. If dropout was greater than or equal to 10%, did the authors assess reasons for dropout or compare those who dropped out to those who remained in follow-up? [d]	TOTAL (N)	TOTAL (%)
Ali (2020) ǂ	1	1	1	0	0	0	1	1	1	1	1	-	-	-	8	73%
Botswana MoH (2013) ǂ	1	1	1	1	0	0	1	1	1	1	1	-	-	-	9	82%
Braunstein (2011)	1	1	1	1	0	0	1	1	1	1	1	1	1	0	11	79%
Chabata (2021)	1	1	1	1	1	1	1	1	1	1	1	1	0	1	13	93%
Chersich (2014)	1	1	0	0	1	1	1	1	1	1	1	1	1	1	12	86%
Diabete (2018)	1	1	1	0	0	1	1	1	1	1	1	1	1	1	12	86%
Faini (2022)	1	1	1	0	0	0	1	1	1	0	0	1	1	0	8	57%
Forbi (2011) ǂ	1	1	0	0	0	0	0	1	1	1	1	-	-	-	6	55%
Fowke (1996)	1	1	1	0	0	0	0	1	1	0	0	1	0	0	6	43%
Ghys (2001)	1	1	1	0	1	0	1	1	0	1	1	1	0	1	10	71%
Gilbert (2003)	1	1	1	0	0	0	1	1	1	1	1	1	0	0	9	64%
Jones (2023)	1	1	1	0	1	0	1	1	1	1	1	1	0	0	10	71%
Kasamba (2019)	1	1	1	0	0	0	1	1	1	1	1	1	1	1	11	79%
Kassanjee (2022) ǂ	1	1	1	1	0	0	1	1	1	1	1	-	-	-	9	82%
Kaul (2004)	1	1	1	1	1	0	0	1	0	1	0	1	1	1	10	71%
Kerrigan (2017)	1	1	1	1	0	0	1	0	0	1	0	1	0	0	7	50%
Kilburn (2018)	1	1	0	0	0	0	0	1	1	1	0	1	0	1	7	50%
Laga (1994)	1	1	0	0	0	0	0	1	1	1	0	1	0	0	6	43%
Lyons (2020)	1	1	1	0	0	0	1	1	1	1	1	1	0	0	9	64%

Malama (2022)	1	1	1	0	1	0	1	1	0	1	0	1	0	0	8	57%
McClelland (2006)	1	1	1	0	0	0	1	1	1	1	1	1	0	1	10	71%
McKinnon (2015)	1	1	1	0	0	0	0	1	0	1	1	1	0	1	8	57%
Nagot (2005)	1	1	0	0	0	0	0	1	0	1	1	1	1	1	8	57%
Naicker (2015)	1	1	1	0	1	1	0	1	1	1	1	1	0	0	10	71%
Nouaman (2022) ‡	1	1	1	0	0	0	0	1	1	1	0	-	-	-	6	55%
Price (2012)	1	1	1	0	1	1	1	1	0	1	1	1	1	1	12	86%
Priddy (2011)	1	1	1	0	0	0	0	1	0	1	1	0	1	0	7	50%
Riedner (2006)	1	1	1	0	0	0	0	1	1	1	1	1	1	1	10	71%
Roddy (1998)	1	1	1	1	1	0	0	1	1	1	0	1	1	1	11	79%
Schim van der Loeff	1	0	1	0	0	0	1	1	1	1	1	1	0	1	9	64%
Thirumurthy (2021)	1	1	1	1	1	1	1	1	1	1	0	1	1	1	13	93%
Van Damme (2002)	1	0	0	1	0	0	0	1	1	1	0	1	0	0	6	43%
TOTAL (N)	32	30	26	9	10	6	19	31	24	30	21	26	12	15		
TOTAL (%)	100%	94%	81%	28%	31%	19%	59%	97%	75%	94%	66%	96%	44%	56%		

a. additional criteria: reporting approach to seroconversion date estimation
b. additional criteria: minimum 12 months follow up
c. additional criteria: 70% retention
d. additional criteria: approaches addressing loss to follow up
‡ Cross-sectional study with adjusted scoring

Supplementary Table S2: Median incidence rate ratios for in women who engage in sex work relative to the national-matched total female population in Eastern and Southern Africa, Western and Central Africa, and Sub-Saharan Africa

Region	Median Incidence Rate Ratio (Inter-quartile Range [IQR])
Eastern and Southern Africa	4·99 (IQR: 3·55-7·02)
Western and Central Africa	22·04 (IQR: 8·22-76·11)
Sub-Saharan Africa	6·08 (IQR: 3·86-9·37)

Supplementary Table S3: Regression model summaries

Covariate	All data, Mean (95% CI)	Kenya Data, Mean (95% CI)	Zimbabwe Data, Mean (95% CI)
**Intercept (Year = 2003)**	1·59 (1·20, 1·99)	1·21 (0·58, 1·80)	0·57 (-0·27, 1·41)
**Year**	-0·02 (-0·09, 0·05)	0·01 (-0·01, 0·02)	0·09 (0·045, 0·14)
**Study Random Intercepts**	3·00 (1·23, 6·30)	1·88 (0·68, 4·07)	8·27 (1·83, 23·72)
**Study Random Slopes with respect to year**	41·98 (17·26, 83·66)		
